# Reconstruction of Panoramic Dental Images Through Bézier Function Optimization

**DOI:** 10.3389/fbioe.2020.00794

**Published:** 2020-07-29

**Authors:** Paulo H. J. Amorim, Thiago F. Moraes, Jorge V. L. Silva, Helio Pedrini, Rui B. Ruben

**Affiliations:** ^1^Tridimensional Technology Division, Center for Information Technology Renato Archer, Campinas, Brazil; ^2^Institute of Computing, University of Campinas, Campinas, Brazil; ^3^CDRsp-ESTG, Polytechnic Institute of Leiria, Leiria, Portugal

**Keywords:** panoramic image, dental arch, computed tomography, diagnostic tool, image reconstruction, Bézier curve

## Abstract

Computed tomography (CT) and X-ray images have been extensively used as a valuable diagnostic tool in dentistry for surgical planning and treatment. Nowadays, dental cone beam CT has been extensively used in dental clinics. Therefore, it is possible to employ three-dimensional (3D) data from the CT to reconstruct a two-dimensional (2D) panoramic dental image that provides a longitudinal view of the mandibular region of the patient, avoiding an additional exposure to X-ray. In this work, we developed a new automatic method for reconstructing 2D panoramic images of the dental arch based on 3D CT images, using Bézier curves and optimization techniques. The proposed method was applied to five patients, some of them with missing teeth, and smooth panoramic images with good contrast were obtained.

## 1. Introduction

Panoramic radiography or panoramic X-ray (Rushton and Horner, 1996; Brennan, 2002; Perschbacher, 2012; Yun et al., 2019) is a two-dimensional (2D) examination used in dentistry to allow for the visualization of the entire dental arch region and adjacent facial structures through only one projection. It provides a useful tool for diagnosis, where upper and lower jaws, teeth, tissues, and surrounding bones are captured in the image.

Computed tomography (CT) is a radiological technique for acquiring three-dimensional (3D) images of body structures. In dental clinics, cone beam CT is commonly used (Bae et al., 2019) since radiation dose is lower when compared to the conventional CT or X-ray modality. The devices are designed in order to perform dental examination with low level of radiation, allowing the analysis of the dental arch region (Langland et al., 2002). Thus, an automatic reconstruction of panoramic images is a important task. This process should also be independent of the acquisition hardware to reduce costs and diagnosis time.

Manual definition of the dental arch region is the gold standard in panoramic image reconstruction (Bae et al., 2019). Therefore, orthodontists or dentists should define a curve, normally a semi-circle, parabola or spline, over the segmented images. This procedure has two main problems: (i) a manual procedure depends on user experience and (ii) a curve, such as a parabola, can be too simple to represent the dental arch. To address these problems, an automatic procedure using Bézier curves formed by a set of points, called control points, is used to determine the shape of the curve. These control point positions are optimized to improve the panoramic image reconstruction.

In the fully automatic process developed in this work, before the Bézier curve definition, it is necessary to load and apply a thresholding segmentation to partition the tooth region. Afterwards, some morphological operators are also used to increase the quality of the mandible and maxilla segmentation. These regions should be connected to define the skeleton and the Bézier curves.

In this work, we present and analyze a method for automatically reconstructing a panoramic radiography from CT images. The proposed approach is based on the use of morphological operators, segmentation techniques, optimization, and filtering methods with low computational cost. Experiments are conducted to demonstrate the effectiveness of the proposed method.

The remainder of this text is organized as follows. In section 2, we review some relevant concepts and approaches related to the topic addressed in our work. In section 3, we present the proposed methodology for automatic reconstruction of panoramic images from CT scans. In section 4, we describe and evaluate the results obtained through the application of the proposed methodology. In section 5, some concluding remarks are presented.

## 2. Background

Medical imaging (Beutel et al., 2000; Hendee and Ritenour, 2003; Bushberg and Boone, 2011; Suetens, 2017) refers to a set of techniques for creating visual representations of the internal organs of a body for clinical purposes, such as disease diagnosis and surgery planning. Procedures, such as X-rays (XR), computed tomography (CT), magnetic resonance imaging (MRI), positron emission tomography (PET), and single-photon emission computed tomography (SPECT) are some examples of diagnostic imaging procedures to diagnose or treat diseases.

More specifically, CT is a radiographic technique that allows the acquisition of volumetric tissue images in regions of interest of the body, facilitating the understanding of its anatomical structures. In dentistry, CT has several applications (Vannier et al., 1984; Abrahams, 1993), for instance, detection and localization of pathologies, planning of implant surgeries, study of facial traumas, reconstruction of tomographic slices of the maxilla, and visualization of bony structures of the lower jaw joint. More recently, CT and cone beam CT have been used for reconstructing panoramic image and other 2D images, such as facial bones, lower jaw regions and nasal cavities (Donker et al., 2002; Taguchi, 2009; Rushton and Rout, 2019). These reconstruction techniques can reduce radiation dose to the patient, when compared to the classic X-ray (Yeh and Chen, 2018).

In the last decades, some procedures have been developed to define a 2D panoramic image from a CT dataset. For instance, Tohnak et al. (2006) calculated the volume projection using the Maximum Intensity Projection (MIP) technique (Wallis et al., 1989), originally called Maximum Activity Projection (MAP). A binary mask in the dental arch of the generated projection is defined by the user. Based on this mask, the center line that horizontally traverses it and divides the image into two parts is calculated. From the middle axis of the image, a Radon transform (Beylkin, 1987) is applied. To generate the panoramic reconstruction, only pixels intercepted by the transform and normal to the tangent of the center line are used. With the use of the Radon transform, the result approximates a panoramic radiography. Their work opened new perspectives in this research topic. However, the proposed method is semi-automatic, since it requires user interaction to select the region of the dental arch.

Gao and Chae (2008) developed an algorithm to find the contact region between lower and upper tooth regions to separate both regions. After separation, a fourth-order polynomial curve fit the dental arch. Integral intensity was calculated along each arch point and used to draw a profile.

Akhoondali et al. (2009) developed a technique to extract panoramic dental images from CT scan images. A segmentation process, with a manual intervention, distinguishes the maxilla and mandible regions by identifying the slice separating them. After this step, a fully automatic procedure using an MIP projection of the slices was applied to isolate the mandible from the other bones. A cubic spline was used to reconstruct the panoramic images.

Bing et al. (2011) presented an automatic procedure to extract panoramic dental images, however, with a high computational cost. They applied a projection of the volumetric image with the MIP technique for automatically selecting the dental arch through the algorithm proposed by Otsu (1979). The center line of the image was obtained with the algorithm developed by Lu and Wang (1986). The reference curve of the dental arch was determined by the least squares method (Bretscher, 1995). The pixels belonging to the obtained curve were selected, providing the panoramic image.

Sa-ing et al. (2013) proposed a fast algorithm for automatic detection of the dental arch. A sequence of synthetic panoramic images and a ray-sum panoramic image were also generated. The authors evaluated their approach on 120 CT axial images.

Amorim et al. (2014) developed a method to automatically reconstruct the dental arch based on CT scan images. Their work employed optimization techniques to find the best B-spline that approximates the dental arch.

Luo et al. (2016) proposed a method to automatically synthesize panoramic dental radiographs from various types of dental cone beam CT data. The dental arch curve was initially generated from the MIP projection. Then, long axial curves of the lower and upper teeth were extracted to created a 3D panoramic curved surface.

More recently, Lee et al. (2019) developed an automatic method to create panoramic dental radiograph from dental cone beam CT images. The approach adaptively found bone HU values on the cone beam CT data. The internal mandible curve was used to synthesize the panoramic radiograph. Yun et al. (2019) also used cone beam CT data to develop an automatic reconstruction with dental arch thickness detection.

In summary, some approaches present automatic reconstruction of panoramic dental images. However, some of them are more focused on dental arch detection, while other ones on the synthesizing process. In the proposed method, an optimization method is used to improve the dental arch definition. A thickness detection algorithm is applied to have a smoother dental arch reconstruction.

## 3. Methodology

The methodology presented in this work is hardware independent, in the sense that the data can be used from different cone beam CT machines. Medical images in the Digital Imaging and Communications in Medicine (DICOM) standard format can be imported to a personal computer and the developed fully automatic method is capable of reconstructing a 2D panoramic dental image. A toolbox was implemented in Python programming language with the NumPy (Numpy, 2014), SciPy (SciPy, 2014), and Visualization Toolkit (VTK) (Schroeder et al., 2006) libraries.

**Figure 1:**
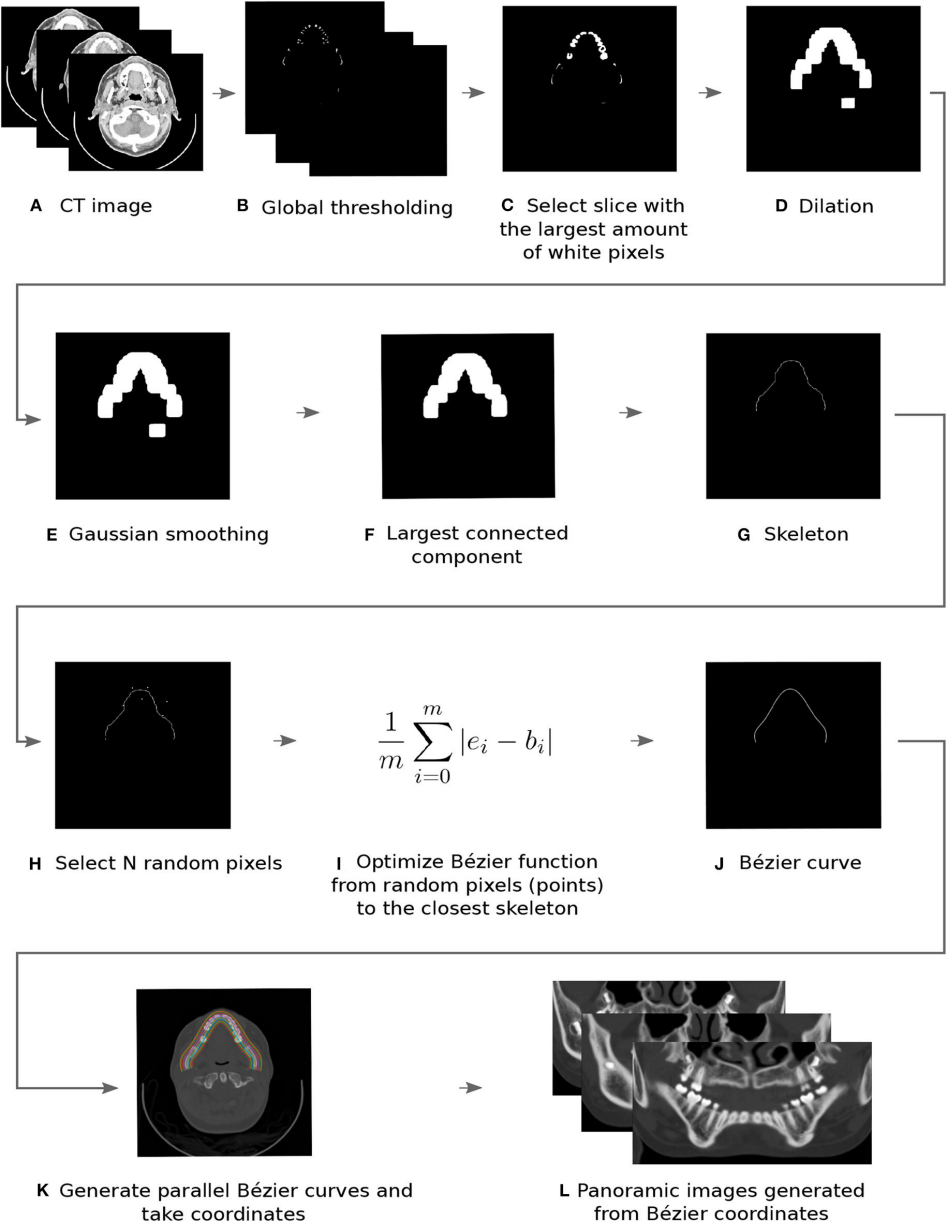
Flowchart of the proposed toolbox from steps **(A–L)**.

The flowchart of the automatic toolbox is presented step by step in Figure 1. After importing the CT images (Figure 1A), a global thresholding segmentation is applied, with a limit value of 1,500 HU (Hounsfield Unit), since dental enamel presents greater intensities in medical images (Figure 1B). The slice with the largest number of segmented voxels is chosen, since this is possibly where the slice with the tip of the teeth is located (Figure 1C).

To connect possible disjoint regions due to, for instance, missing teeth, a morphological dilation operator of size 20 × 20 pixels was applied (Amorim et al., 2012, 2014). The result, presented in Figure 1D, is not smooth enough to define the skeleton. Therefore, in the next step, a Gaussian filter with σ = 4 is used (Amorim et al., 2012, 2014). In Figure 1E, a smoother image is observed, however, more than one component is still visible. Then, the largest related object is chosen, as illustrated in Figure 1F.

In this stage, it is possible to define the skeleton, which is a curve that passes through the middle axis of the largest connected component (defined in the previous step). To do so, a medial axis algorithm was implemented (Dey, 2006) and the skeleton curve was generated and presented in Figure 1G.

The skeleton is not a smooth curve. In fact, first, second, or third derivatives are not continuous functions. For this reason, a panoramic image based on skeleton will be non-smooth (Amorim et al., 2014). Thus, a smooth curve should be applied. In manual processes, a parabola is the most commonly used, since it is easy to implement. However, it is not possible to accurately define the dental arch with a parabola (Bae et al., 2019).

Polynomial curves are a natural choice to define the dental arch, since they are smooth (Piegl and Tiller, 2012). A Bézier curve (Hughes et al., 2013) is a polynomial curve of degree *n* defined by *n* control points (*P*_*i*_):

(1) $$\begin{array}{r} B(t)=\sum_{i=0}^{n}\left(\begin{array}{c} n\\ i \end{array}\right){\left(1-t\right)}^{n-i}t^{i}P_{i} \end{array}$$

where *t* is the curve parameter and ranges from 0 to 1. The closer to 1, the closer to the end of the curve the resulting point will be. In a Bézier curve of degree *n*, the first *n* derivatives are all continuous, so a Bézier curve is *C*^*n*^ (Piegl and Tiller, 2012).

To increase the panoramic image quality, it is also important to define a curve as close as possible to the skeleton curve. In fact, it is possible to propose an optimization procedure to compute control points (*P*_*i*_) that minimize the distance between the Bézier and skeleton curves. Mathematically, the optimization procedure can be stated as (Amorim et al., 2014):

(2) $$\begin{array}{r} \min f\left(P_{i}\right)=\frac{1}{\Gamma_{a}}\int_{\Gamma_{a}}\Phi \Gamma \end{array}$$

where Γ_*a*_ is the skeleton curve length coordinate, whereas Φ represents the distance between both curves, Bézier and skeleton. However, since the image is expressed in a discrete representation, it is not possible to apply a continuous function. Therefore, the optimization function must be adapted to a discrete analysis:

(3) $$\begin{array}{r} \min f\left(P_{i}\right)=\frac{1}{m}\overset{m}{\underset{i=0}{\sum }}\Phi =\frac{1}{m}\overset{m}{\underset{i=0}{\sum }}|e_{i}-b_{i}| \end{array}$$

where *b*_*i*_ is the *i*-th point of the Bézier curve and *e*_*i*_ is the *i*-th point of the skeleton, which is the closest one from *b*_*i*_ point. Value *m*+1 is the number of points where distance is computed. In this case, *m* must be a large number and *m* = 1, 000 was used in our work. In order to obtain optimized Bézier curves, the Sequential Least Squares Programming (SLSQP) (Fiacco and McCormick, 1990) method was employed. This method is available on SciPy library (SciPy, 2014). The optimization sequence, from the initial step where *n* random control points are chosen, to the optimized Bézier curve, is presented in Figures 1H–J.

Dental arch is defined by the optimized Bézier curve. However, it is necessary to define the dental arch thickness. Therefore, since the *B*(*t*) curve is found, parallel *CP*_*i*_ curves can be calculated. For this, the derivative (Equation 4) is calculated for each point of the *B*(*t*) curve and rotated 90° counterclockwise and spaced by a *q* offset.

(4) $$\begin{array}{r} {\text{B}}^{\prime }\left(t\right)=n\overset{n-1}{\underset{i=0}{\sum }}b_{i,n-1}\left(t\right)\left({\text{P}}_{i+1}-{\text{P}}_{i}\right) \end{array}$$

After calculating the *B*(*t*) and *CP*_*i*_ curves (Figure 1K), pixels that intersect each curve are extracted for each slice (Figure 1L). The final panoramic 2D image is a synthesized image obtained with Average Intensity Projection (AIP) (Da Re et al., 2019) projection that was applied to each extracted slice.

## 4. Experimental Results

For validation purposes, the fully automatic methodology proposed to reconstruct panoramic dental images was applied to five cone beam CT images from different patients. Two patients have missing teeth. For each patient, tests were also conducted using 3, 7, 11, and 15 control points. Main characteristics of the cone beam CT data are presented in Table 1.

**Table 1:** Main characteristics of the data sets.

**Patient**	**Number of slices**	**Dimension (pixels)**
A	99	512 × 512
B	240	512 × 512
C	252	512 × 512
D	198	512 × 512
E	177	512 × 512

The cost function (Equation 2) evolution for all different cases is presented in Table 2. For 3 control points defining the Bézier curve, the cost function is always smaller than 5.71 (4.94 on average). The Bézier curve defined by 3 control points is a parabola. In this case, however, parabolas minimize the distance between the skeleton and dental arch curves. In the manual dental arch definition, a parabola is commonly used. Therefore, in a perfect situation, manually defined parabolas are equal to the optimized ones. From the visualization of the 3-control point curves in **Figure 3**, it is possible to observe that the Bézier curve (orange) does not overlap the tooth in an important dental region.

**Table 2:** Cost function.

**Patient**	**3 control points**		**7 control points**		**11 control points**		**15 control points**	
	***f_initial_***	***f_final_***	***f_initial_***	***f_final_***	***f_initial_***	***f_final_***	***f_initial_***	***f_final_***
A	203.19	5.70	203.37	2.59	203.18	1.60	203.23	0.87
B	91.48	5.20	91.44	3.02	91.48	1.40	91.47	0.72
C	133.11	4.08	133.18	1.23	133.19	0.83	133.13	0.61
D	129.71	5.27	129.68	2.08	129.80	1.30	129.84	0.91
E	193.80	4.49	193.82	1.76	193.69	1.23	193.75	0.68

**Figure 2:**
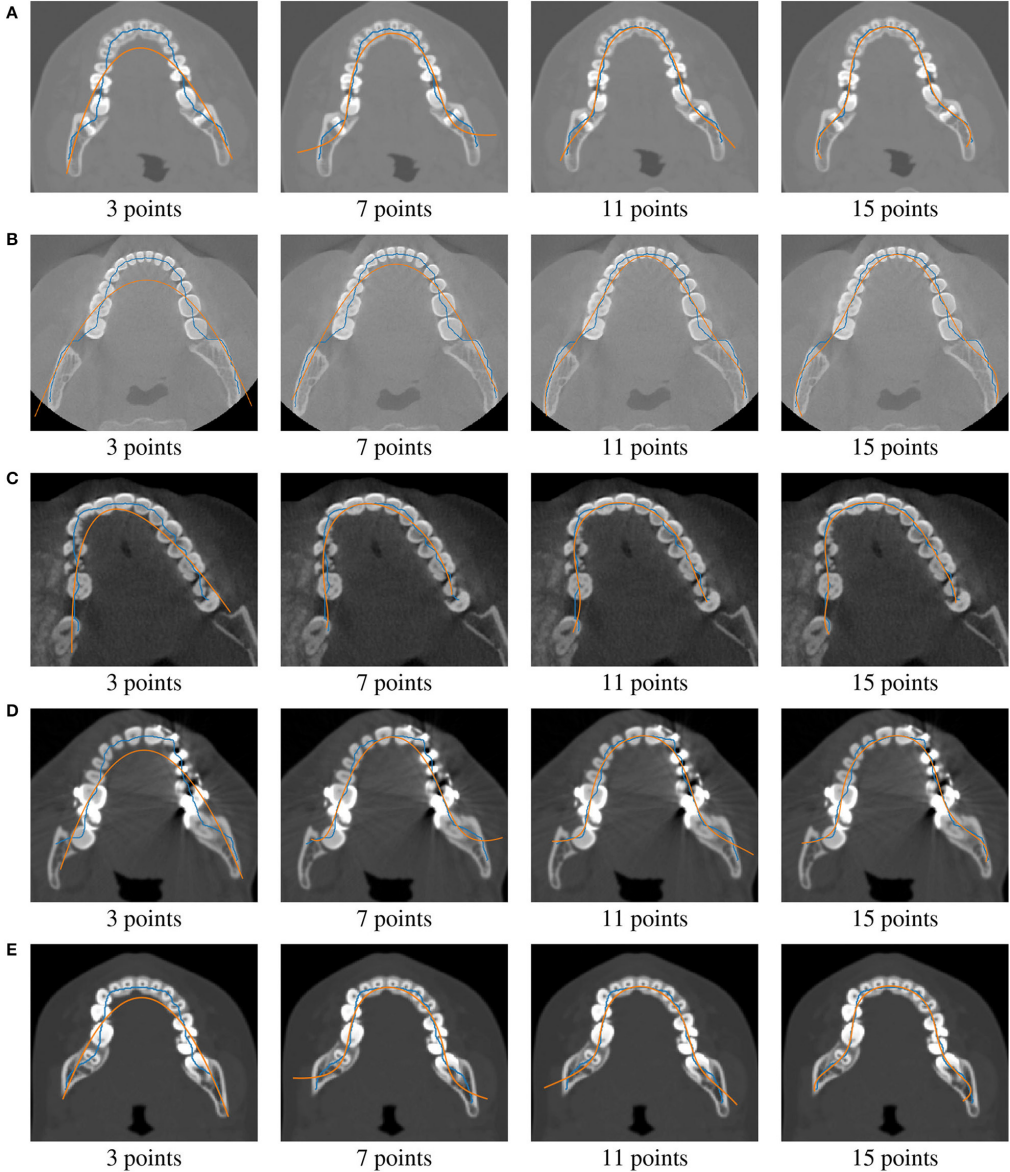
Application of our methodology to Patients **(A–E)** (from top to bottom rows). Skeleton curves are shown in blue color, whereas optimized Bézier curves are shown in orange color.

For 7 control points, the cost function is always smaller than 2.60 (2.13 on average). In this case, the Bézier curve (in orange color) does not sometimes over all the dental region (Figure 2). However, the Bézier curve is now closer to the skeleton curve (in blue color). For 11 and 15 control points, the cost function is smaller than 1.61 (1.27 on average) and 0.92 (0.75 on average), respectively. In both cases, Bézier curves overlap tooth on all dental regions. The Bézier curve (in orange color) is very close to the skeleton curve (in blue color). These results show that 11 and 15 control points are better when compared to 3 and 5 points. In Figure 2, it is also possible to observe that the skeleton curves are not smooth.

**Figure 3:**
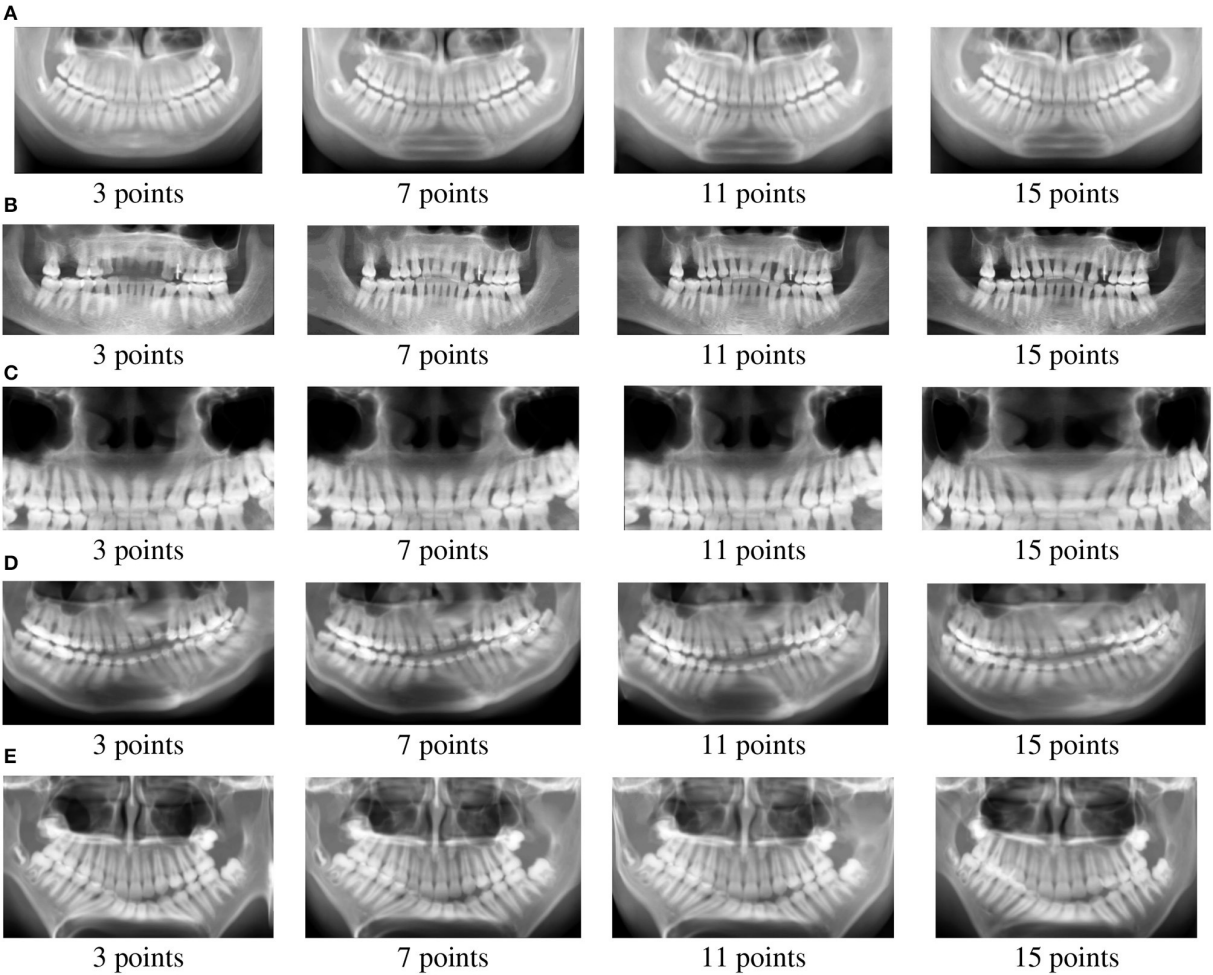
Panoramic images for Patients **(A–E)** (from top to bottom rows).

The panoramic images extracted from the Bézier curves are illustrated in Figure 3. As expected, the panoramic images obtained with 3 control points have missing information. For instance, some teeth in the upper jaw are not well-defined for patient B. Considering 7 points, all teeth are present in the panoramic images, however, images are blur and tooth root details are more difficult to observe. For 11 and 15 control points, the generated panoramic images are very similar, at least in a macro analysis. In fact, the panoramic images with 11 and 15 control points are softer and present more details than the images generated considering 3 and 7 control points. Average Intensity Projection (AIP) (Da Re et al., 2019) projection was applied to the set of extracted panoramic images.

## 5. Conclusions

This work presented a fully automatic method for the reconstruction of panoramic dental images from a set of cone beam CT images of patients using Bézier curves combined with optimization techniques.

According to the experimental results, the proposed methodology has demonstrated to be very effective, serving as an important computational tool in the dentistry field. The developed toolbox is user-friendly and its computational cost is low, since the reconstruction is performed only once for each patient. For the optimization procedure, it is possible to define a smooth curve with a small amount of control points. Optimized Bézier curves with 11 and 15 points can be a proper strategy for generating panoramic dental images.

Since the Bézier curve limits (end and start) are difficult to specify (Piegl and Tiller, 2012), as illustrated in Figure 2, it would be important to evaluate the proposed method through other parametric curves to approximate the dental arch. The proposed toolbox should also be applied to more patients with different pathologies.

## Data Availability Statement

The original contributions presented in the study are included in the article/supplementary material, further inquiries can be directed to the corresponding author/s.

## Author Contributions

All authors listed have made a substantial, direct and intellectual contribution to the work, and approved it for publication.

## Conflict of Interest

The authors declare that the research was conducted in the absence of any commercial or financial relationships that could be construed as a potential conflict of interest.
